# Sensor-Based Quantification of MDS-UPDRS III Subitems in Parkinson’s Disease Using Machine Learning

**DOI:** 10.3390/s24072195

**Published:** 2024-03-29

**Authors:** Rene Peter Bremm, Lukas Pavelka, Maria Moscardo Garcia, Laurent Mombaerts, Rejko Krüger, Frank Hertel

**Affiliations:** 1 National Department of Neurosurgery, Centre Hospitalier de Luxembourg, 1210 Luxembourg, Luxembourghertel.frank@chl.lu (F.H.); 2 Parkinson’s Research Clinic, Centre Hospitalier de Luxembourg, 1210 Luxembourg, Luxembourg; lukas.pavelka@lih.lu (L.P.); rejko.krueger@lih.lu (R.K.); 3 Translational Neuroscience, Luxembourg Centre for Systems Biomedicine, University of Luxembourg, 4365 Esch-sur-Alzette, Luxembourg; 4 Transversal Translational Medicine, Luxembourg Institute of Health, 1445 Strassen, Luxembourg; 5 Systems Control, Luxembourg Centre for Systems Biomedicine, University of Luxembourg, 4365 Esch-sur-Alzette, Luxembourg

**Keywords:** Parkinson’s disease, motor symptoms, MDS-UPDRS, wearable devices, inertial measurement unit, machine learning, random forest

## Abstract

Wearable sensors could be beneficial for the continuous quantification of upper limb motor symptoms in people with Parkinson’s disease (PD). This work evaluates the use of two inertial measurement units combined with supervised machine learning models to classify and predict a subset of MDS-UPDRS III subitems in PD. We attached the two compact wearable sensors on the dorsal part of each hand of 33 people with PD and 12 controls. Each participant performed six clinical movement tasks in parallel with an assessment of the MDS-UPDRS III. Random forest (RF) models were trained on the sensor data and motor scores. An overall accuracy of 94% was achieved in classifying the movement tasks. When employed for classifying the motor scores, the averaged area under the receiver operating characteristic values ranged from 68% to 92%. Motor scores were additionally predicted using an RF regression model. In a comparative analysis, trained support vector machine models outperformed the RF models for specific tasks. Furthermore, our results surpass the literature in certain cases. The methods developed in this work serve as a base for future studies, where home-based assessments of pharmacological effects on motor function could complement regular clinical assessments.

## 1. Introduction

Parkinson’s disease (PD) is currently the fastest-growing neurodegenerative disease in the world, clinically defined by a typical triad of motor symptoms, including bradykinesia, extrapyramidal rigidity, and/or rest tremor. Currently, over 10 million people worldwide are affected by PD, and this number is expected to increase substantially to over 17 million people by 2040 [1,2]. To date, however, no disease-modifying interventions have been identified, and therapy is limited to symptomatic and supportive treatment. Moreover, the long-term use of dopaminergic treatment, in parallel with disease progression, eventually leads to motor fluctuations, imposing a significant burden on patients and caregivers in terms of quality of life [3,4,5].

Assessing the severity of symptoms and progression in PD is crucial for managing the disease effectively [6,7]. Typically, symptoms are scored by a trained physician employing the Movement Disorder Society (MDS) Unified Parkinson’s Disease Rating Scale (UPDRS) [8]. Part III of the MDS-UPDRS serves as the most common standard assessment tool for quantifying motor symptoms in PD. The scale consists of 18 items rating rigidity, bradykinesia, tremor, speech, posture, gait, and balance. Each item is rated on a 5-point scale with separate scores assigned to each side of the body. The scale corresponds as follows—0 for no symptoms, 1 for slight, 2 for mild, 3 for moderate, and 4 for severe impairment. MDS-UPDRS III requires patients to perform various activities and movement tasks to assess symptoms such as tremor and bradykinesia [8,9,10]. While standardised training is offered for this scale, healthcare practitioners may still vary in their scoring of patients, resulting in intra-rater and inter-rater variability, which may impact on the reliability and reproducibility [11,12]. Furthermore, this qualitative method is labour- and resource-intensive when scoring each symptom by an expert in movement disorders [12,13].

The use of wearable technology to characterise clinical features has the potential to significantly improve the reliability, reproducibility, and accessibility of motor symptom assessment in PD [14,15,16,17]. Passive monitoring systems, for example, could autonomously assess motor symptoms through regular task-specific movements at home to reduce bias in subjective scoring [18,19,20]. Additionally, those systems could decrease the frequency of in-person clinic visits while offering a more comprehensive insight into symptoms throughout the day, thereby optimising treatment strategies and the management of PD symptoms [7,16,21]. Objective methods proposed to assess bradykinesia and tremor often employ accelerometer and gyroscope sensors [22,23,24,25], mobile devices (e.g., smartphones/watches) [26,27], and video-based motion capture systems [28,29,30,31]. In a recent study, finger tapping (MDS-UPDRS item 3.4) was assessed in 37 people with PD using index finger accelerometry [19]. An open-source tool was then developed for the automated assessment of bradykinesia. The tool detected tapping blocks in over 94% of cases and predicted motor scores correlated positively with expert ratings in over 70% of cases [19]. In another PD study, motor scores for hand resting tremor (MDS-UPDRS item 3.17) were predicted with 85.5% accuracy using a custom-built wearable device assembled with an accelerometer and a gyroscope [32]. Aside from inertial sensors, smartphone-based methods for capturing finger-tapping tasks have shown reliable correlations with MDS-UPDRS motor scores [26,33]. Similarly, video-based recordings of movements are useful for predicting expert-rated MDS-UPDRS motor scores [34,35]. Their reliance on patient self-recording and lack of external validation, however, limits comparability and reproducibility [34].

One of the key challenges, apart from collecting sufficient labelled data to model the manifestations of motor symptoms [36], is to objectively assess motor symptoms based on all relevant clinical movement tasks in people with PD. In addition, achieving this with a minimal number of unobtrusive wearable sensors for future home monitoring solutions aims to address the practical challenges associated with resource-intensive clinical assessments [13,18]. This could ease time constraints in clinical settings and reduce healthcare costs. To move towards this goal, the focus of this feasibility study is to develop an objective method for assessing motor scores (MDS-UPDRS III scores) on all relevant arm and hand movement tasks (MDS-UPDRS III tasks). Each hand was equipped with a single, compact, wearable inertial measurement unit (IMU) sensor consisting of a 3-axis accelerometer, a 3-axis gyroscope, and a 3-axis magnetometer. Despite the accessibility and relatively low cost of wearable motion sensors, to the best of our knowledge, there are no published methods for classifying and predicting motor scores across six clinically structured movement tasks using a single wearable motion sensor attached to each hand. Therefore, we assessed the performance of two compact wearable IMUs in combination with supervised machine learning (ML) models for classifying and predicting MDS-UPDRS III scores for six MDS-UPDRS III tasks.

## 2. Materials and Methods

### 2.1. Study Population and Study Design

Clinical and sensor-based data were collected during the regular study visits of participants in the Luxembourg Parkinson’s study. This nationwide, monocentric, observational, and prospective cohort study has been recruiting and is following up patients with PD, all other forms of atypical parkinsonism, and controls longitudinally. To this moment, more than 1600 participants have been recruited, comprising more than 800 patients with PD or atypical parkinsonism and more than 800 controls. Controls were defined as individuals above 18 years without signs of a neurodegenerative disorder, active cancer, or pregnancy. The detailed recruitment strategy, diagnostic criteria, and inclusion/exclusion criteria of the Luxembourg Parkinson’s study were described extensively in Hipp et al., 2018 [37]. For this study, a population of 33 patients with PD according to the UK PD Brain Bank Criteria [38] and 12 controls were selected based on their willingness to participate in the sensor assessment during regular sequential visits. No additional exclusion and inclusion criteria were applied. The group of 33 PD patients covers all motor-symptom-related subtypes, including 14 mixed subtypes, 13 kinetic-rigid subtypes, and 2 tremor-predominant subtypes. In four patients, the subtypes were not specified. Ethical approval for data and sample collection and written informed consent were obtained from all participants. Demographic and clinical characteristics of both study groups are shown in Table 1.

Participants were seated in a chair with a backrest (and no armrests) and were asked to perform six MDS-UPDRS III tasks related to arm and hand movements (data collection protocol in Supplementary Material, Table S1). The descriptions of the visually guided movements are shown in Table 2, including Arm at Rest (AR), Outstretched Arm (OA), Finger to Nose (FN), Hand Movement (HM), Pronation/Supination (PS), and Finger Tapping (FT). Patients performed the tasks as quickly as possible with fingers spread as wide as possible. Each movement task was performed for at least 10 s with two repetitions, and motor symptoms were assessed by the study physician in the medication ON condition. AR, OA, and FN were additionally repeated by performing a dual task where the MDS-UPDRS III task was combined with a subtracting numbers aloud task. The dual task served to amplify the underlying tremor, if present, in the task AR, OA, and FN with the corresponding rating by the study physician. All rating conditions were compliant with the MDS-UPDRS III instructions.

### 2.2. Sensor Setup

Following the aim of this feasibility study to develop an objective method for scoring motor symptoms, a compact wearable sensor prototype (miPod v1, Portabiles HealthCare Technologies GmbH, Erlangen, Germany) was attached to each participant’s hand using small strips of adhesive tape. The hardware platform of the sensor device contains various electronic components and is assembled with a 9-axis IMU consisting of a 3-axis accelerometer (16-bit, setting ±8 G), 3-axis gyroscope (16-bit, setting ±2000 degrees per second), and 3-axis magnetometer (13-bit, ±1200 µT). miPod weighs about 12 g, and its polymer case has a size of approximately (35 × 25 × 8) mm in length, width, and thickness [39]. The sensor devices were placed in the centre of the back of each hand to measure the participants’ movements during the execution of the tasks AR, OA, FN, HM, and PS. To measure participants’ movement during the FT task, the wearable IMUs were then attached to the intermediate phalanges of each index finger. Placing the sensors on the dorsal part of each hand has been proven to be effective for training symptom detection models for bradykinesia and tremor in PD [36].

### 2.3. Data Processing

IMU sensor data were recorded at a frequency of 200 Hz, read out from the internal sensor memory (1 GB NAND flash) via Micro USB cable, and analysed offline with custom-written software in Python^TM^ (Version 3.8.8, Python Software Foundation, Wilmington, DE, USA) and MATLAB^TM^ (R2022a, MathWorks Inc., Natick, MA, USA). The data were downsampled to 50 Hz to reduce data size and improve computational efficiency. This sampling rate has proven to be suitable for analysing human body movements measured by accelerometers [40]. A visual inspection of the spectral components of the measured arm and hand movements revealed that those movements contained frequency components below 20 Hz. Dimensionality was reduced by calculating the Euclidean norm (signal magnitude) for each sensor type (i.e., accelerometer, gyroscope, and magnetometer). A digital Butterworth lowpass filter (3-pole IIR, 10 ms delay) with a cut-off frequency of 20 Hz was then applied to each time series data of the 9-axis IMU.

Each movement task was recorded for approximately 10 s. An epoch of 5 s was then manually isolated from each recording (centre position) to remove unstable signal components on both sides. Next, each epoch was divided into two equal parts to capture movement variability within each segment, as bradykinesia typically manifests as a progressive reduction in speed and amplitude of repetitive movements toward the end of a clinical task [41].

### 2.4. Feature Extraction

Time and frequency domain features were computed for each data segment to train supervised ML models for classification and prediction tasks. The defined set of features corresponds to a recently published study with similar objectives [13]. Welch’s power spectral density was estimated to compute frequency domain features within a range of 0 to 10 Hz. Table 3 shows the set of features that were computed for each half-segment. All features were standardised by removing their mean and scaling them to the unit variance (z-score).

### 2.5. Machine Learning Models

Random forest (RF) ML models were developed and trained on the magnitude features (Table 3) of each sensor type for both classification and regression tasks. The model type was chosen due to its ability to handle imbalanced datasets and its advantages, such as high performance, a low number of hyperparameters, and the ability to reduce overfitting [42]. Support vector machine (SVM) models were implemented for comparative analysis, and both RF and SVM models were additionally trained on all axis features of the IMU. These results can be found in Supplementary Tables S2–S6 and Figures S5 and S6, including an overview of all trained models in Table S8.

Four RF models were developed for certain purposes. Each movement task was treated as a distinct class in each of the models. The first model aimed to distinguish between the movement tasks, as outlined in Table 2. In contrast to this multiclass approach, the second model was designed as a binary classifier to differentiate between patients and controls with a motor score of zero and patients with a non-zero motor score. Similar to the first approach, the third model also employed a multiclass solution with a focus on classifying patients’ non-zero motor scores. Unlike the previous three models, the fourth model used an RF regression technique. This model was built to predict individual non-zero motor scores.

To optimise the performance of the RF models, a grid search approach was employed. A range of possible parameters was manually specified for hyperparameter tuning. The hyperparameters considered for tuning included the number of estimators (trees, 10:5:150), the maximum depth of trees (5, 16, 28, 40), the minimum samples required to split an internal node (2, 5, 10), and the minimum samples required for leaf nodes (1, 2, 4). Bootstrapping was enabled. Selected hyperparameters for both model types can be found in the Supplementary Section hyperparameters and in Table S7. To assess the performance of the RF models with dependable hyperparameters, repeated stratified k-fold cross-validation was employed, with fours fold repeated five times. This method ensures that each fold’s class distribution is representative of the entire dataset, mitigating a potential bias introduced by imbalanced classes.

As a performance metric, receiver operating characteristic (ROC) curves were computed for each classification model, and the averaged area under the ROC (AUROC) values were calculated to provide an overview of overall performance. Note that a balanced dataset was used for classifying MDS-UPDRS III tasks, and the overall accuracy was computed as a performance metric. Boxplots were employed to visualise the prediction of individual MDS-UPDRS III scores.

## 3. Results

A total of 540 measurements were performed on 45 participants. Although each participant performed the six clinically structured tasks on each hand (Table 2), 32 tasks spread across 6 participants (1 control and 5 PD) were not accurately recorded due to sensor-related issues. Corresponding records from these contaminated tasks were removed. This resulted in an imbalanced dataset of 508 tasks for 45 participants for motor score classification and prediction. The classification of the movement tasks was performed on a balanced dataset (subset) consisting of 28 people with PD and 11 controls. The 39 participants represent a complete dataset in which six movement tasks were accurately recorded on each hand.

**Figure 1:**
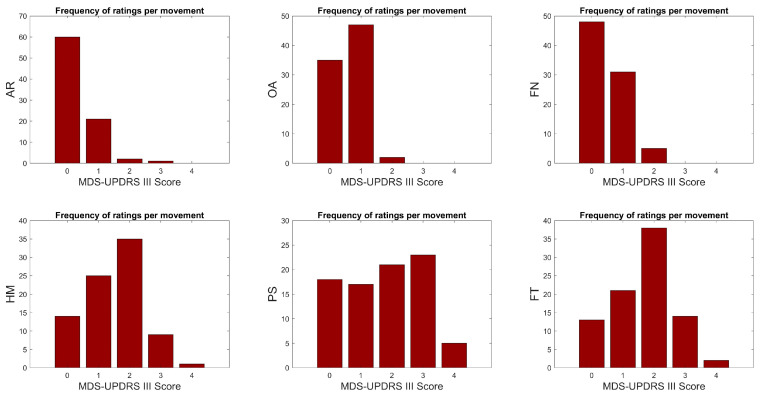
Frequency of motor score ratings for each movement task. The number of motor score {0,1,2,3,4} ratings of 45 participants were for Arm at Rest (AR) {60,21,2,1,0}, Outstretched Arm (OA) {35,47,2,0,0}, Finger to Nose (FN) {48,31,5,0,0}, Hand Movement (HM) {14,25,35,9,1}, Pronation/Supination (PS) {18,17,21,23,5}, and Finger Tapping (FT) {13,21,38,14,2}. Note that the data set only contains controls with motor score {0}.

The frequency of motor score ratings for each MDS-UPDRS III task is illustrated for all participants in Figure 1. Movement tasks AR, OA, and FN do not possess ratings of motor score 4, while OA and FN do not possess ratings of motor score 3. Given the significant imbalance in the dataset, certain motor score categories were excluded for classification purposes. Specifically, motor scores 2 and 3 in AR, motor score 2 in OA, motor score 4 in HM, and motor score 4 in FT were omitted from the analysis to ensure a more balanced and manageable dataset.

**Figure 2:**
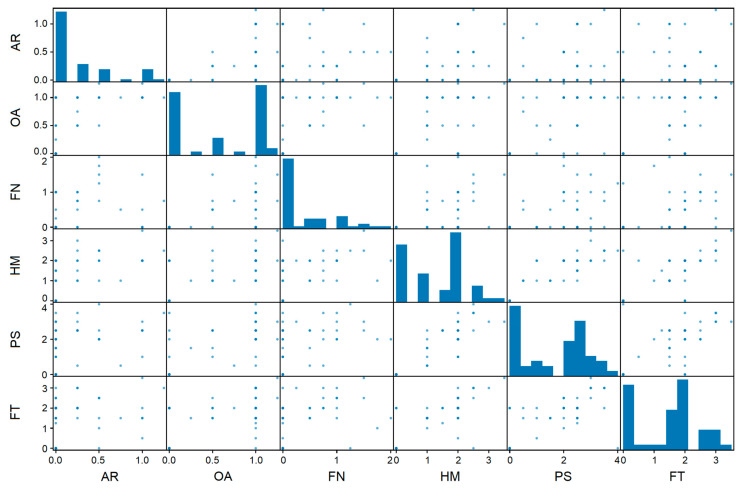
Correlations between movement tasks and motor symptom ratings. To illustrate how patient ratings relate to different movements, the average rating was calculated separately for each patient and each movement, resulting in intermediate values in this scatter plot matrix. The plot visualises the variability in symptom expression, and patients may experience different levels of severity within each task, as reflected in the MDS-UPDRS III subitems.

The relationship between motor symptom ratings and MDS-UPDRS III tasks in the context of patient-specific variability is visualised in the scatter plot matrix in Figure 2. The diagonal elements show individual histograms for each MDS-UPDRS III task, illustrating the frequency distribution of motor symptom ratings specific to each task. In contrast, the off-diagonal elements show scatter plots highlighting the correlations between motor score ratings associated with different movements for each patient. Correlations emerged between certain subsets of patients, for example, for the HM and FT tasks, indicating that these two movements may share common symptom characteristics.

### 3.1. Classification of MDS-UPDRS III Tasks

The performance of the RF model in classifying movement tasks is shown in the confusion matrix in Table 4. The model correctly classified the six MDS-UPDRS III tasks with an overall accuracy of 94.2%. An overall accuracy of 90.3% was achieved with the SVM model (Supplementary Table S2).

### 3.2. Classification of MDS-UPDRS III Scores

To assess the classification performance of each RF model, the averaged AUROC values with corresponding 95% confidence intervals (CIs) were calculated.

For binary classification, Table 5 shows the averaged AUROC values for distinguishing between patients and controls with a motor score of zero {0} and patients with a non-zero motor score {1,2,3,4}. Averaged AUROC values with the best result in each MDS-UPDRS III task (marked in bold in each task column, Table 5) ranged from 72% to 92%.

**Figure 3:**
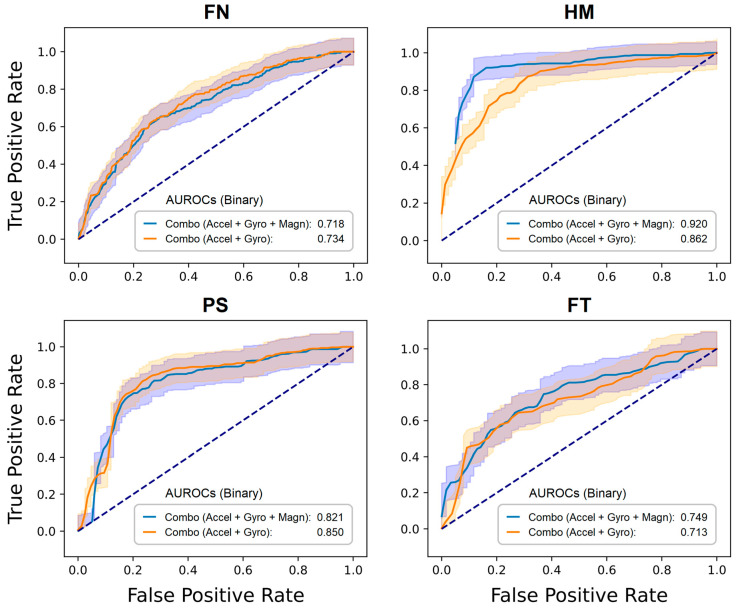
ROC curves to distinguish between zero and non-zero motor scores. Random forest models were trained on the magnitude features derived from the data of the two sensor configurations commonly found in IMUs offered by manufacturers. The averaged area under the ROC (AUROC) values (with 95% CIs, shaded area) refer to Table 5.

The SVM models outperformed the RF models in Table 5 for the tasks listed in Supplementary Table S3 (SVM models trained on sensor magnitude features) for PS (89%); Table S4 (RF models trained on sensor axis features) for AR (74%), HM (93%), and FT (82%); and Table S6 (SVM models trained on sensor axis features) for HM (94%) and FT (90%). The results in Table 5 are comparable to those observed in the literature and occasionally surpass them [13,19,32,36]. A detailed description of how our results surpass those in the literature can be found in the Discussion section and in Table S9 (Supplementary Material). Moreover, the classification performance varies depending on both the type of movement and the sensor employed, either individually or in combination. For example, gyroscope data alone are critical for the HM tasks; however, they offer highly relevant information for PS. In addition to the results in Table 5, four ROC curves for the two sensor configurations commonly found in IMUs are shown in Figure 3. The ROC curves of AR and OA are not depicted in the figure as they do not involve active MDS-UPDRS III tasks. For instance, in AR, both forearms and hands are resting on the lap, and in OA, both arms and hands are outstretched (posture). FN and FT in Figure 3 show comparable AUROC performance for both sensor combinations. Conversely, the HM and PS tasks displayed a modest enhancement in classification accuracy when the three sensors were combined.

For multiclass classification, Table 6 shows the averaged AUROC values for classifying non-zero motor scores {1,2,3,4} in patients with PD. Averaged AUROC values with the best result in each MDS-UPDRS III task (marked in bold in each task column, Table 6) ranged from 68% to 85%. Performances in Table 6 decreased compared to the binary classification in Table 5, reflecting challenges in the multiclass classification task. The SVM models outperformed the RF models for the tasks listed in Supplementary Table S5 (RF models trained on sensor axis features) with HM (77%) and PS (79%).

### 3.3. Prediction of MDS-UPDRS III Scores

**Figure 4:**
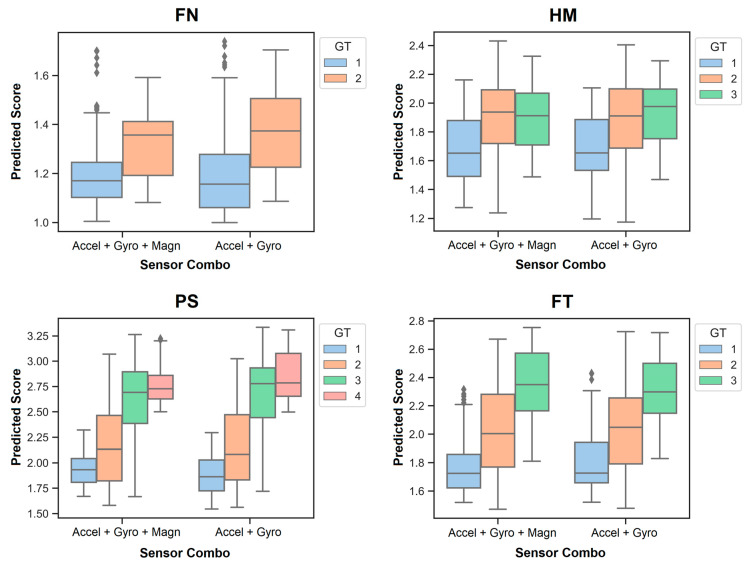
Prediction of individual motor scores in PD.

RF regression models were developed to predict the individual motor scores {1,2,3,4} of the patients with PD for two sensor configurations commonly found in IMUs. Figure 4 shows the results, wherein the legends illustrate the motor score ratings given by the study physician. Those are referred to as ‘ground truth’ (GT) labels. The continuous numeric values on the vertical axis of each graph in the figure display the prediction outcomes of an RF regression model. Boxplots illustrate the accuracy, variability, and overlap of predictions for each motor score across MDS-UPDRS III tasks. Both sensor combinations in each graph, as shown in Figure 4, demonstrate comparable prediction performance. It should be noted that the predicted motor scores closest to the GT scores are those that occur most frequently in the dataset for each MDS-UPDRS III task (Figure 1).

Random forest regression models were trained on the magnitude features derived from the data of the two sensor configurations commonly found in IMUs offered by manufacturers. The box plots illustrate the accuracy, variability, and overlap of the predictions for each motor score across the MDS-UPDRS III tasks. Motor scores closest to the ‘ground truth’ (GT) score were the most frequent ones in each MDS-UPDRS III task.

## 4. Discussion

### 4.1. Overview of Results

In this feasibility study, a compact wearable IMU sensor was positioned on each hand to develop an objective method for assessing motor scores in PD. To the best of our knowledge, there has been no method published to date using our approach, specifically for the classification and prediction of MDS-UPDRS III subitems (motor scores) across six MDS-UPDRS III tasks. Data collection protocols were optimised to reduce the time for setting up the sensors and carrying out the measurements. On average, each participant devoted six minutes to complete the six MDS-UPDRS III tasks on both hands. Overall, this implemented setting has proven to be a more practical and feasible solution for collecting sensor data in a dynamic and fast-paced clinical environment. Sensor-based time domain and spectral features were computed for model training. RF was chosen as the model type due to its ability to handle imbalanced datasets. SVM was added for comparative analysis (Supplementary Material). The six MDS-UPDRS III tasks were correctly classified with an average accuracy of 94.2%. Averaged AUROC values, with the highest results for each MDS-UPDRS III task, varied between 72% and 92% for distinguishing between zero and non-zero motor scores and between 68% and 85% for classifying non-zero motor scores in the context of PD. The classification performance varied depending on the type of sensor employed. Motor scores were additionally predicted using an RF regression model. However, comparing predicted individual motor scores with GT labels remains a challenging task.

### 4.2. Comparison with Previous Work

Previous studies using accelerometers and gyroscopes have mainly focused on specific clinical tasks to predict motor scores, such as placing the hands on the lap to assess hand resting tremor or performing the finger-tapping test to assess bradykinesia, focusing on decrement in rate, amplitude, or both with repetitive action [19,32,43]. Results presented in our study (e.g., AUROC curves) are comparable to those observed in the literature and occasionally surpass them [13,19,32,36]. For example, two studies investigated the effect of sensor placement and combinations of inertial sensors on symptom detection (tremor and bradykinesia) [13,36]. Binary and multiclass RF models were employed in those studies. In another two studies, motor scores were classified and predicted, with each focusing on a single movement task [19,32]. Different aspects from those four studies are integrated into our work for the classification and prediction of motor scores from all relevant arm and hand movements according to MDS-UPDRS III. A detailed comparison can be found in Table S9 (Supplementary Material).

More recently, machine learning solutions that focus on markerless video-based motion capture technologies have been introduced to analyse human body movements in clinical settings [28,29,35]. However, ethical concerns about the protection of patients’ privacy arise in studies where video data is recorded, in particular for continuous video monitoring at home [44]. In addition, accurate detection and tracking of specific landmarks are critical to the success of markerless motion tracking systems, as they form the basis for understanding and analysing the subject’s movements in video data [31,45]. Complex movements involve rapid changes in position and orientation, which can lead to the occlusion of landmarks. Similarly, changes in camera angles, distances, and lighting conditions (e.g., shadows or reflections) might affect the visibility of landmarks. Upcoming research could compare sensor-based machine learning models in contrast with video-based markerless tracking models for the quantification of MDS-UPDRS III subitems.

### 4.3. Interpretability of Results

Hand movement recognition is critical for a self-managed home system to automatically assess upper limb motor function in patients with PD. To move toward this objective, we have developed an ML model to classify MDS-UPDRS III tasks. This potentially streamlines the process of automatically generating more training data in the future, where patients with PD could be encouraged to perform exercise programs at home for remote assessment of motor symptom severity by using compact wearable sensors [46]. Building on this, we have additionally developed ML models to classify and predict MDS-UPDRS III scores. Following this two-step approach, our aim was to simplify the data measurement and processing pipeline by using two compact IMUs, reducing the data sampling rate to 50 Hz, and employing a suitable set of features for model training.

Based on a similar recently published study [13], these criteria drain the battery and data storage of the sensors and might reduce the computational cost for model training. Although our selection of a limited set of simple and interpretable features was informed by a recent study [13], it seems evident that these features may not capture the entire complexity of human body movements. Conversely to the limited set of features, each sensor of the IMU maps a unique, measurable phenomenon associated with motor symptoms that are consistent with the observations of the study physician. Furthermore, we divided the sensor data into two equal segments for feature extraction. This division aimed to capture movement variability in each segment, as motor symptoms typically expose a progressive reduction in either the frequency or amplitude of repetitive movements toward the end of a clinical task [47]. For example, a bradykinesia-induced decrement in fine motor control of the hands might cause poor performance in daily routine activities, such as brushing teeth or handwriting [13]. We often observed this decrement in rate and amplitude with repetitive action in the FT task, and it partially correlated with the motor scores given by the study physician.

### 4.4. Limitations

Despite considerable efforts to produce high-quality data in a clinical setting, collecting more data for training would presumably improve the accuracy of our machine learning models, as indicated in our results. To build our study cohort, we followed an initial estimate to derive a similar number of patients as reported in previous research studies [13,19,36]. It is further important to note that the number of controls and their age profiles were not specifically matched to those of the patient group. The dataset created reflects real-world scenarios sourced from routine clinical visits. Patients attended the clinical assessments alongside their accompanying person (e.g., spouse), who volunteered as a control. Another aspect to consider is the observed divergence in gender within and between groups and their potential impact on the internal validity of the study. Moreover, the training data collected in this study were part of a standardised motor assessment [8]. It is important to note that the results of a study on activity recognition in stroke patients using mobile phones have demonstrated that models trained on patients’ activities performed in a clinical setting may not generalise well to activities performed at home [46]. Although the MDS-UPDRS III tasks were designed to closely mimic natural behaviour [8], it is still critical to validate the performance of any symptom detection and severity prediction model during the day-to-day activities of people with PD [7,23].

Placing a single sensor on each hand may not capture the entire information about the severity of a motor symptom in PD. In contrast, a previous study showed that a single wearable motion sensor placed on the back of each hand was sufficient to detect upper limb motor symptoms [36]. Additionally, wearable devices integrated with an accelerometer and gyroscope, designed to be worn unobtrusively on the body (e.g., integrated into the shoe, belt, or wrist), offer the potential to generate robust, reliable, and reproducible data, both within and across individuals [20,36]. They could further aid physicians in saving both effort and time by enabling comparative analysis and ensuring consistent monitoring of the progression of motor impairments in PD. Recent studies have shown that digital biomarkers of disease severity have been integrated as valid surrogate biomarkers in various clinical trials [48,49].

Moreover, the discrepancies between our model predictions and the GT scores defined by the study physician may indicate nuances in the consistency of subjective motor symptom assessments [50]. One aspect to consider here is the fact that a major limitation across any subitem of the MDS-UPDRS is the arbitrary categorisation of an otherwise continuous phenomenon. The sensor information appears to be more granular in terms of subtle movements compared to this arbitrary categorisation by motor score {0,1,2,3,4} based on a complex combination of visual assessment and visual impression. The sensor measurements reflect the underlying subtle changes in movement, which may or may not be evident in the assessment made by the trained study physician. In future studies, an additional rater could be included. However, this does not resolve the issue when the two raters differ by only one point. An intermediate motor score is not accounted for in the MDS-UPDRS.

### 4.5. Future Work

Our findings demonstrate the potential of machine learning models in predicting disease severity based on clinical sensor data. The methods developed and models trained in this work serve as a foundation for future studies, where pharmacological effects on motor function could complement regular clinical motor assessments. To move toward this objective, we will focus on expanding our data cohort by incorporating new training datasets to capture a more comprehensive view of patients’ motor symptoms, potentially enhancing the predictive capabilities of our models. As part of this effort, we aim to modify our MDS-UPDRS III task classifier for real-time applications. The adapted classifier will then be implemented into TreCap, our custom-built wearable device, which is equipped with MATLAB software (R2022a) for real-time sensor data acquisition and the management of visually guided arm and hand movements [51]. In addition, the study’s reliance on a limited set of simple and interpretable features may overlook the complexity of motor symptoms and their manifestations in PD patients. Hence, we might consider employing more complex features and feature selection methods to identify optimal features for each motor symptom in PD for model training [52].

As a performance metric, we chose AUROC as it captures the performance of our models across all possible decision thresholds. While we have evaluated model performance using AUROC, upcoming research could explore whether different metrics (e.g., F1 score, positive predictive value, among others) can reproduce our results for the classification and prediction of MDS-UPDRS III subitems. Moreover, we aim to compare our methods with video-based markerless tracking models. Finally, the use of deep learning models could be explored on a larger data cohort in the future.

## Figures and Tables

**Table 1:** Demographic and clinical characteristics of study participants.

	PD (n = 33)	Controls (n = 12)
Age at assessment (years, mean ± SD)	69.8 ± 8.7	60.8 ± 9.8
Sex (numbers male/female)	29/4	1/11
Time since diagnosis (years mean ± SD)	5.2 ± 4.6	Not applicable
Hoehn and Yahr Scale (mean ± SD) *	2.2 ± 0.7	0
MDS-UPDRS I total score (mean ± SD)	9.1 ± 4.6	7.9 ± 7.3
MDS-UPDRS II total score (mean ± SD)	10.5 ± 7.1	1.3 ± 2.8
MDS-UPDRS III total score (mean ± SD) *	40.9 ± 12.7	4.8 ± 3.5
MDS-UPDRS IV total score (mean ± SD)	1.5 ± 2.8	0
Total sum of MDS-UPDRS I-IV (mean ± SD)	61.9 ± 19.0	14.1 ± 10.9
Montreal Cognitive Assessment (mean ± SD) *	24.1 ± 3.5	27 ± 2.8
Levodopa Equivalent Daily Dose (mean ± SD)	499.1 ± 459.4	Not applicable
Time since last L-DOPA intake (minutes mean ± SD)	155.9 ± 113.1	Not applicable

* Assessment in medication ON state for PD.

**Table 2:** Subset of MDS-UPDRS III tasks performed by participants during the visits of the assessment of PD symptoms.

Movement	Label	Description of the Movement	MDS-UPDRS Reference
Task 1	AR	Forearms/hands rest on lap	3.17.1 and 3.17.2
Task 2	OA	Outstretched arms and hands with spread fingers	3.15.1 and 3.15.2
Task 3	FN *	Nose touching via index finger	3.16.1 and 3.16.2
Task 4	HM	Opening and closing the palm of the hands	3.5.1 and 3.5.2
Task 5	PS	Arm supination and pronation (aligned with hands)	3.6.1 and 3.6.2
Task 6	FT	Tapping between thumb and index finger	3.4.1 and 3.4.2

* This task begins and ends with forearms and hands resting on the lap.

**Table 3:** Features categorisation for ML models.

Feature Category	Features	Number of Tri-Axial Features	Number of Magnitude * Features
Time	Root mean square, range, mean, variance, skew, kurtosis	18	6
Frequency	Dominant frequency, relative magnitude, moments of power spectral density (mean, standard deviation, skew, kurtosis)	18	6
Entropy	Sample entropy	3	1
Total for each sensor type		39	13

* The features were derived from the magnitude signals (Euclidean norm) of each sensor type within the IMU.

**Table 4:** Performance of a random forest model trained on all magnitude features within the IMU for MDS-UPDRS III tasks classification.

Tasks		Predicted Class						True Positive and Negative Rate
		AR	OA	FN	HM	PS	FT	
True class	AR	68	9	0	1	0	0	0.872
	OA	5	73	0	0	0	0	0.936
	FN	1	0	75	0	1	1	0.962
	HM	0	0	1	75	1	1	0.962
	PS	0	0	1	2	75	0	0.962
	FT	0	0	1	1	1	75	0.962
Positive and negative predictive value		0.919	0.890	0.962	0.949	0.962	0.974	Accuracy * 0.942

* Multiclass classification performance: Sensitivity 0.942, Specificity 0.988, Precision 0.943, F1-Score 0.942, Matthews Correlation Coefficient 0.930, and Cohen’s Kappa 0.931. Operating points on ROC curves with AUROC values: 0.982 (AR), 0.989 (OA), 0.997 (FN), 0.997 (HM), 0.997 (PS), and 0.998 (FT).

**Table 5:** Performance of random forest models trained on the magnitude features of each sensor type. Averaged AUROC values (with 95% CIs) for distinguishing between patients and controls with a motor score of zero and patients with a non-zero motor score.

Sensor *	MDS-UPDRS III Tasks **					
	AR	OA	FN	HM	PS	FT
Accelerometer	0.65 (0.59–0.73)	0.75 (0.68–0.82)	0.66 (0.59–0.74)	0.84 (0.76–0.92)	0.51 (0.40–0.62)	0.67 (0.57–0.77)
Gyroscope	0.71 (0.64–0.78)	0.77 (0.71–0.85)	0.70 (0.63–0.77)	0.72 (0.63–0.83)	0.82 (0.74–0.91)	**0.77** (0.68–0.86)
Magnetometer	0.57 (0.50–0.65)	0.64 (0.56–0.72)	0.63 (0.55–0.71)	0.87 (0.80–0.95)	0.51 (0.41–0.63)	0.73 (0.64–0.83)
Accel + Gyro	0.69 (0.62–0.76)	0.77 (0.71–0.85)	**0.73** (0.66–0.80)	0.86 (0.79–0.94)	**0.85** (0.77–0.93)	0.71 (0.62–0.81)
Accel + Magn	0.69 (0.62–0.76)	0.75 (0.68–0.82)	0.68 (0.61–0.76)	0.90 (0.85–0.97)	0.51 (0.41–0.63)	0.73 (0.64–0.83)
Gyro + Magn	0.70 (0.64–0.77)	0.77 (0.71–0.84)	0.68 (0.62–0.76)	0.90 (0.84–0.97)	0.80 (0.72–0.89)	0.72 (0.63–0.82)
Accel + Gyro + Magn	**0.72** (0.65–0.79)	**0.78** (0.71–0.85)	0.72 (0.65–0.79)	**0.92** (0.86–0.98)	0.82 (0.74–0.90)	0.75 (0.66–0.84)

* Performance for each sensor type and all sensor combinations. Binary classification with motor scores {0} vs. {1} for AR and OA tasks, {0} vs. {1,2,3} for FN, HM, and FT tasks, and {0} vs. {1,2,2,3,4} for PS tasks. ** AUROC values with the best result in each column are highlighted in bold.

**Table 6:** Performance of random forest models trained on the magnitude features of each sensor type. Averaged AUROC values (with 95% CIs) for classifying patients with non-zero motor scores.

Sensor *	MDS-UPDRS III Tasks **			
	FN	HM	PS	FT
Accelerometer	**0.85** (0.79–0.91)	0.56 (0.45–0.67)	0.69 (0.59–0.79)	0.61 (0.50–0.72)
Gyroscope	0.76 (0.69–0.83)	0.55 (0.44–0.66)	**0.73** (0.63–0.83)	0.65 (0.55–0.75)
Magnetometer	0.52 (0.44–0.60)	**0.68** (0.58–0.78)	0.54 (0.43–0.65)	0.70 (0.60–0.80)
Accel + Gyro	0.83 (0.77–0.89)	0.58 (0.47–0.69)	0.70 (0.60–0.80)	0.66 (0.56–0.76)
Accel + Magn	0.82 (0.76–0.88)	0.62 (0.51–0.73)	0.66 (0.56–0.76)	0.70 (0.60–0.80)
Gyro + Magn	0.66 (0.58–0.74)	0.61 (0.50–0.72)	0.72 (0.62–0.82)	0.66 (0.56–0.76)
Accel + Gyro + Magn	0.81 (0.75–0.87)	0.61 (0.50–0.72)	0.73 (0.63–0.83)	**0.71** (0.61–0.81)

* Performance for each sensor type and all sensor combinations. Multiclass classification with motor scores {1,2,3} for FN, HM, and FT tasks, and {1,2,2,3,4} for PS tasks. ** AUROC values with the best result in each column are highlighted in bold.

## Data Availability

The datasets generated and analysed in this study are not publicly available due to national and institutional regulations at the Parkinson’s Research Clinic. However, the data are available upon reasonable request in compliance with those regulations. Requests should be referred to request.ncer-pd@uni.lu. The underlying code for this study is available upon request.

## Supplementary Materials

The following supporting information can be downloaded at https://www.mdpi.com/article/10.3390/s24072195/s1, Figure S1: ROC curves to distinguish between zero and non-zero motor scores. Random forest models were trained on all axis features extracted from the data of two sensor configurations commonly found in IMUs offered by manufacturers. The averaged area under the ROC (AUROC) values (with 95% CIs, shaded area) refer to Table S4; Figure S2: Prediction of individual motor scores in PD. Random Forest regression models were trained on the magnitude features derived from the data of the two sensor configurations commonly found in IMUs offered by manufacturers. The box plots illustrate the accuracy, variability, and overlap of the predictions for each motor score across the MDS-UPDRS III tasks. Motor scores closest to the ’ground truth’ (GT) score were the most frequent ones in each MDS-UPDRS III task; Table S1: Data collection protocol. The standardised clinical motor assessments were conducted by all study participants after their routine clinical examinations in the National Centre of Excellence in Research on Parkinson’s Disease at the Centre Hospitalier de Luxembourg. Date and time of a measurement are contained in the ’ND number’ and ’Number of Visit’ information; Table S2: Performance of a support vector machine model trained on all magnitude features within the IMU for MDS-UPDRS III tasks classification; Table S3: Performance of support vector machine models trained on the magnitude features of each sensor type. Averaged AUROC values (with 95 % CIs), for distinguishing between patients and controls with a motor score of zero and patients with a non-zero motor score; Table S4: Performance of random forest models trained on all axis features of each sensor type. Averaged AUROC values (with 95 % CIs), for distinguishing between patients and controls with a motor score of zero and patients with a non-zero motor score; Table S5: Performance of random forest models trained on all axis features of each sensor type. Averaged AUROC values (with 95 % CIs), for classifying patients with non-zero motor scores; Table S6: Performance of support vector machine models trained on all axis features of each sensor type. Averaged AUROC values (with 95 % CIs), for distinguishing between patients and controls with a motor score of zero and patients with a non-zero motor score; Table S7: Hyperparameters of the random forest models in Table 5; Table S8: An overview of all trained machine learning models in this study; Table S9: Comparison with studies in the literature.
