# Supplementary material for: Sensor-Based Quantification of MDS-UPDRS III Subitems in Parkinson’s Disease Using Machine Learning
Source: Sensors (Basel). 2024 Mar 29;24(7):2195. doi: 10.3390/s24072195 (PMC11014392; doi:10.3390/s24072195)
Supplement: Supplementary file 1 [file sensors-24-02195-s001.zip › sensors-2916958-supplementary.pdf]

SUPPLEMENTARY MATERIAL

S1. Data collection protocol.

ND number:		Number of Visit:		
Minutes since the last dose of L-DOPA:			UPDRS part III	
Subject group (Control or PD):			Right	Left
**Task 1**	AR, Forearms/hands rest on lap, relaxed	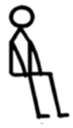		
Task 1*	AR, Forearms/hands rest on lap (math 96-5) (tremor-related patients only)			
**Task 2**	OA, Outstretched arm (posture), relaxed	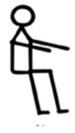		
Task 2*	OA, Outstretched arm (posture), provoked (math 83-9) (tremor-related patients only)			
**Task 3**	FN, Finger to nose (5 times), relaxed	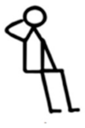		
Task 3*	FN, Finger to nose (5 times), provoked (math 88-6) (tremor-related patients only)			
**Task 4**	HM, Hand movement (open-close), relaxed	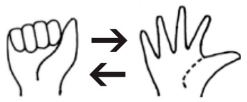		
**Task 5**	PS, Pronation-Supination (rotation), relaxed	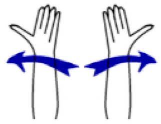		
**CHANGE IN POSITION OF THE SENSORS:**				
**Task 6**	FT, Finger tapping, relaxed	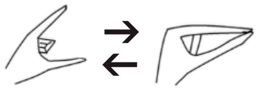		
Notes:				

Table S1. Data collection protocol. The standardised clinical motor assessments were conducted by all study participants after their routine clinical examinations in the National Centre of Excellence in Research on Parkinson's Disease at the Centre Hospitalier de Luxembourg. Date and time of a measurement are contained in the 'ND number' and 'Number of Visit' information.
*Subtraction of numbers aloud in addition to the movement task to amplify the underlying tremor in task AR (rest tremor), task OA (postural tremor) and task FN (kinetic tremor).

S2. Classification performances.

SVM models trained on all magnitude features within the IMU (Comparative analysis to Table 4):

Table S2. Performance of a support vector machine model trained on all magnitude features within the IMU for MDS-UPDRS III tasks classification.

**Tasks**		**Predicted class**						True positive and negative rate
		**AR**	**OA**	**FN**	**HM**	**PS**	**FT**	
**True class**	AR	67	10	0	1	0	0	0.859
	OA	12	65	0	1	0	0	0.833
	FN	1	0	76	1	0	0	0.974
	HM	3	1	0	74	0	0	0.949
	PS	0	0	2	2	74	0	0.949
	FT	0	0	0	4	0	74	0.949
Positive and negative predictive value		0.807	0.855	0.974	0.892	1.000	1.000	Accuracy* 0.903

*Multiclass classification performance: Sensitivity 0.919, Specificity 0.983, Precision 0.919, F1-Score 0.920, Matthews Correlation Coefficient 0.903, and Cohen's Kappa 0.903. Operating points on ROC curves with AUROC values: 0.969 (AR), 0.982 (OA), 0.991 (FN), 0.994 (HM), 0.998 (PS), and 0.999 (FT).

SVM trained on magnitude features of each sensor type (Comparative analysis to Table 5):

Table S3. Performance of support vector machine models trained on the magnitude features of each sensor type. Averaged AUROC values (with 95 % CIs), for distinguishing between patients and controls with a motor score of zero and patients with a non-zero motor score.

**Sensor***	**MDS-UPDRS III tasks****					
	**RP**	**OA**	**FN**	**HM**	**PS**	**FT**
Accelerometer	0.66 (0.59-0.73)	0.71 (0.63-0.78)	0.67 (0.60-0.75)	0.77 (0.68-0.87)	0.47 (0.36-0.58)	**0.72** (0.62-0.82)
Gyroscope	**0.67** (0.60-0.74)	0.58 (0.50-0.66)	0.68 (0.61-0.75)	0.44 (0.32-0.55)	0.87 (0.80-0.94)	0.44 (0.33-0.55)
Magnetometer	0.52 (0.45-0.60)	0.46 (0.38-0.54)	0.48 (0.40-0.56)	0.87 (0.80-0.95)	0.39 (0.28-0.50)	0.33 (0.22-0.43)
Accel + Gyro	0.61 (0.54-0.68)	**0.73** (0.66-0.80)	**0.69** (0.62-0.77)	0.88 (0.81-0.95)	**0.89** (0.82-0.96)	0.61 (0.51-0.72)
Accel + Magn	0.65 (0.58-0.72)	0.66 (0.58-0.73)	0.62 (0.54-0.70)	0.85 (0.78-0.93)	0.41 (0.31-0.52)	0.64 (0.54-0.75)
Gyro + Magn	0.67 (0.60-0.74)	0.48 (0.39-0.56)	0.66 (0.58-0.73)	**0.91** (0.84-0.97)	0.81 (0.73-0.90)	0.75 (0.66-0.84)
Accel + Gyro + Magn	0.62 (0.55-0.70)	0.69 (0.61-0.76)	0.66 (0.58-0.73)	0.88 (0.81-0.95)	0.85 (0.77-0.93)	0.70 (0.60-0.80)

*Performance for each sensor type and all sensor combinations. Binary classification with motor scores {0} vs. {1} for AR and OA tasks, {0} vs. {1,2,3} for FN, HM, and FT tasks, and {0} vs. {1,2,2,3,4} for PS tasks.
** AUROC values with the best result in each column are highlighted in bold.

RF models trained on all axis features of each sensor type (Comparative analysis to Table 5):

Table S4. Performance of random forest models trained on all axis features of each sensor type. Averaged AUROC values (with 95 % CIs), for distinguishing between patients and controls with a motor score of zero and patients with a non-zero motor score.

**Sensor***	**MDS-UPDRS III tasks****					
	**RP**	**OA**	**FN**	**HM**	**PS**	**FT**
Accelerometer	**0.74** (0.67-0.81)	0.74 (0.68-0.81)	0.67 (0.59-0.74)	0.85 (0.77-0.93)	0.80 (0.72-0.89)	0.80 (0.72-0.89)
Gyroscope	0.72 (0.65-0.79)	**0.77** (0.70-0.83)	0.69 (0.62-0.76)	0.88 (0.80-0.95)	**0.85** (0.77-0.93)	0.74 (0.64-0.83)
Magnetometer	0.52 (0.44-0.59)	0.56 (0.48-0.64)	0.65 (0.56-0.72)	0.92 (0.86-0.98)	0.62 (0.51-0.73)	0.80 (0.71-0.88)
Accel + Gyro	0.74 (0.68-0.81)	0.76 (0.70-0.83)	**0.71** (0.64-0.79)	0.86 (0.79-0.94)	0.85 (0.77-0.93)	0.79 (0.70-0.88)
Accel + Magn	0.71 (0.64-0.78)	0.75 (0.71-0.84)	0.68 (0.60-0.75)	**0.93** (0.87-0.98)	0.70 (0.59-0.60)	0.70 (0.60-0.88)
Gyro + Magn	0.70 (0.63-0.77)	0.76 (0.70-0.83)	0.67 (0.60-0.75)	0.92 (0.85-0.98)	0.83 (0.75-0.91)	0.81 (0.72-0.89)
Accel + Gyro + Magn	0.73 (0.66-0.79)	0.77 (0.70-0.84)	0.70 (0.62-0.77)	0.92 (0.86-0.98)	0.85 (0.77-0.93)	**0.82** (0.74-0.91)

*Performances for each sensor type and all sensor combinations. Binary classification with motor scores {0} vs. {1} for AR and OA tasks, {0} vs. {1,2,3} for FN, HM, and FT tasks, and {0} vs. {1,2,2,3,4} for PS tasks.
** AUROC values with the best result in each column are highlighted in bold.

In addition to the results in **Table S10**, four ROC curves are shown in **Figure S5.**

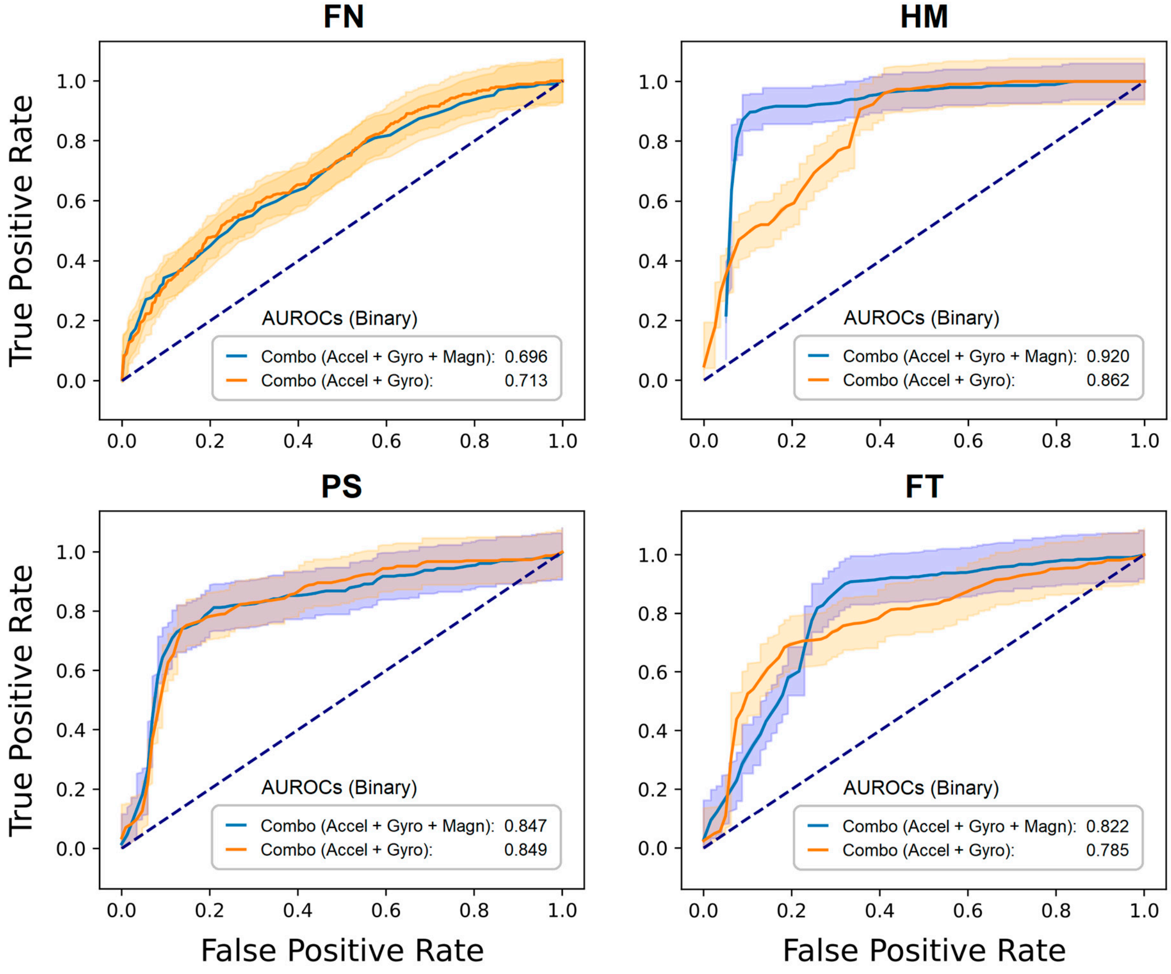

Figure S1. ROC curves to distinguish between zero and non-zero motor scores. Random forest models were trained on all axis features extracted from the data of two sensor configurations commonly found in IMUs offered by manufacturers. The averaged area under the ROC (AUROC) values (with 95% CIs, shaded area) refer to Table 10.

RF models trained on all axis features of each sensor type (Comparative analysis to Table 6):

Table S5. Performance of random forest models trained on all axis features of each sensor type. Averaged AUROC values (with 95 % CIs), for classifying patients with non-zero motor scores.

**Sensor***	**MDS-UPDRS III tasks****			
	**FN**	**HM**	**PS**	**FT**
Accelerometer	**0.77** (0.70-0.83)	0.62 (0.51-0.73)	0.74 (0.68-0.87)	0.65 (0.55-0.75)
Gyroscope	0.72 (0.65-0.80)	0.52 (0.41-0.63)	0.78 (0.69-0.87)	**0.71** (0.62-0.81)
Magnetometer	0.52 (0.44-0.60)	0.68 (0.58-0.78)	0.54 (0.43-0.65)	0.70 (0.60-0.80)
Accel + Gyro	0.77 (0.70-0.84)	**0.77** (0.68-0.87)	**0.79** (0.70-0.88)	0.63 (0.52-0.73)
Accel + Magn	0.77 (0.70-0.83)	0.60 (0.49-0.70)	0.66 (0.56-0.76)	0.67 (0.56-0.77)
Gyro + Magn	0.73 (0.66-0.80)	0.60 (0.49-0.71)	0.74 (0.64-0.84)	0.71 (0.61-0.81)
Accel + Gyro + Magn	0.74 (0.67-0.81)	0.60 (0.50-0.71)	0.75 (0.65-0.84)	0.71 (0.61-0.81)

*Performance for each sensor type and all sensor combinations. Multiclass classification with motor scores {1,2,3} for FN, HM, and FT tasks, and {1,2,2,3,4} for PS tasks.
** AUROC values with the best result in each column are highlighted in bold.

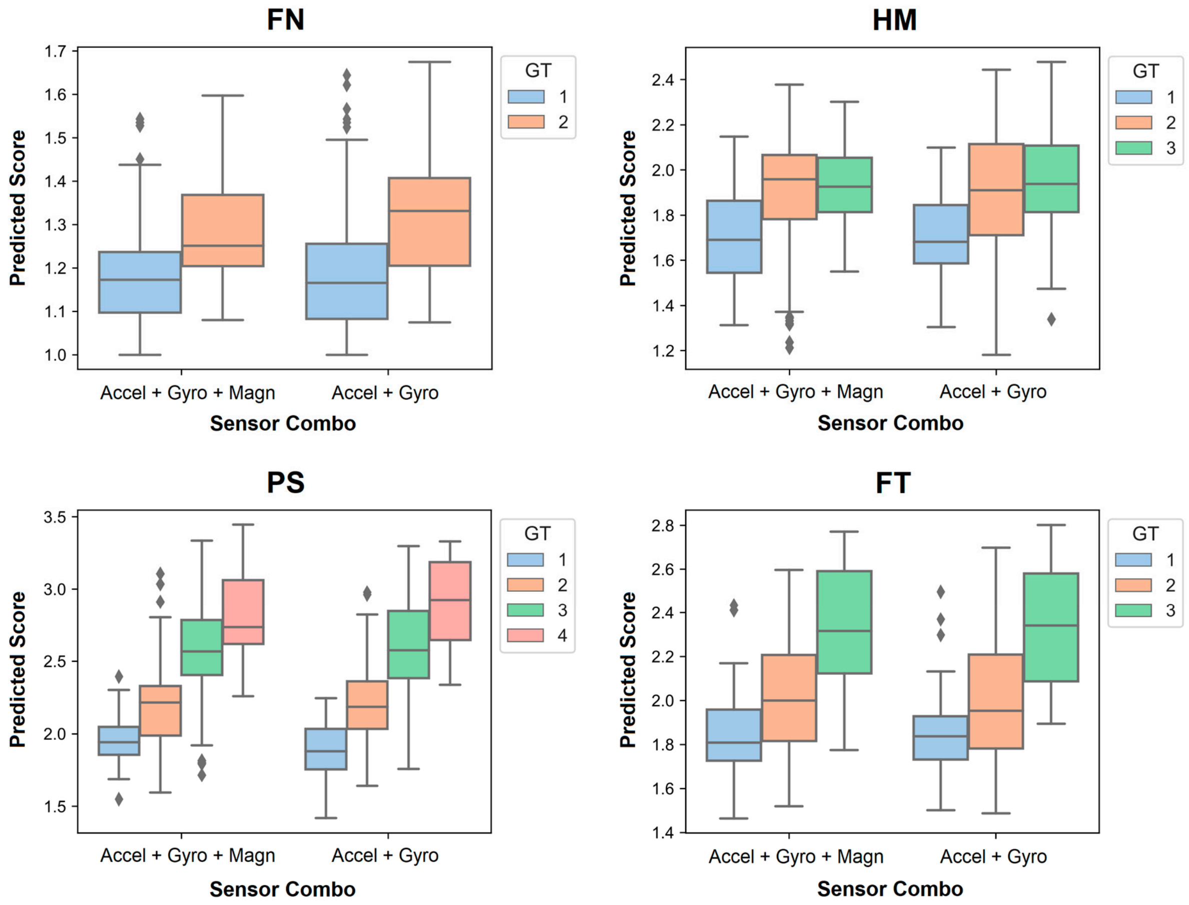

Figure S2. Prediction of individual motor scores in PD. Random Forest regression models were trained on the magnitude features derived from the data of the two sensor configurations commonly found in IMUs offered by manufacturers. The box plots illustrate the accuracy, variability, and overlap of the predictions for each motor score across the MDS-UPDRS III tasks. Motor scores closest to the 'ground truth' (GT) score were the most frequent ones in each MDS-UPDRS III task.

SVM models trained on all axis features of each sensor type (Comparative analysis to Table 6):

Table S6. Performance of support vector machine models trained on all axis features of each sensor type. Averaged AUROC values (with 95 % CIs), for distinguishing between patients and controls with a motor score of zero and patients with a non-zero motor score.

**Sensor***	**MDS-UPDRS III tasks****					
	**RP**	**OA**	**FN**	**HM**	**PS**	**FT**
Accelerometer	0.65 (0.58-0.72)	**0.61** (0.53-0.79)	**0.73** (0.66-0.80)	0.86 (0.78-0.93)	0.39 (0.28-0.50)	0.88 (0.81-0.95)
Gyroscope	0.69 (0.62-0.76)	0.56 (0.48-0.64)	0.62 (0.55-0.70)	0.89 (0.82-0.96)	0.81 (0.72-0.90)	0.49 (0.38-0.60)
Magnetometer	0.56 (0.48-0.63)	0.48 (0.40-0.56)	0.61 (0.53-0.69)	**0.94** (0.89-0.99)	0.64 (0.53-0.74)	0.33 (0.22-0.43)
Accel + Gyro	**0.71** (0.64-0.78)	0.63 (0.55-0.70)	0.71 (0.64-0.78)	0.91 (0.85-0.98)	**0.84** (0.76-0.92)	0.82 (0.73-0.90)
Accel + Magn	0.54 (0.47-0.62)	0.60 (0.42-0.58)	0.70 (0.63-0.78)	0.94 (0.89-0.99)	0.50 (0.39-0.61)	**0.90** (0.83-0.96)
Gyro + Magn	0.67 (0.60-0.74)	0.50 (0.42-0.58)	0.71 (0.64-0.78)	0.93 (0.87-0.98)	0.79 (0.73-0.90)	0.83 (0.75-0.92)
Accel + Gyro + Magn	0.66 (0.59-0.73)	0.56 (0.49-0.64)	0.71 (0.64-0.79)	0.93 (0.87-0.98)	0.78 (0.69-0.87)	0.87 (0.80-0.94)

*Performance for each sensor type and all sensor combinations. Binary classification with motor scores {0} vs. {1} for AR and OA tasks, {0} vs. {1,2,3} for FN, HM, and FT tasks, and {0} vs. {1,2,2,3,4} for PS tasks.
** AUROC values with the best result in each column are highlighted in bold.

S3. Hyperparameters in random forest models.

A grid search was performed using cross-validation with a repeated k-fold stratification method (four folds, repeated five times) to optimise the hyperparameters for each RF model. This process explored various combinations, including the number of trees, maximum depth and minimum samples required for node splitting. The best hyperparameter combinations were determined based on model performance, improving the predictive accuracy of the random forest classifier on unseen data.

The grid search started with the following random parameter setting:
(1) Number of trees in the tested forest with values: 10:5:150.
(2) Square root function considered for the total number of features at each split.
(3) Maximum depth of trees tested with values: 5, 16, 28, and 40.
(4) Minimum number of samples required to split a node: 2, 5 and 10.
(5) Minimum number of samples required at each leaf node: 1, 2 and 4.
(6) Method used to select samples for building and training each tree: Bootstrap.

Results of a grid search with optimal hyperparameters in Tabel 5 (example):

Table S7. Hyperparameters of the random forest models in Table 5.

**Description of hyperparameters**	**Optimal hyperparameters***					
	**AR**	**OA**	**FN**	**HM**	**PS**	**FT**
Maximum depth of the decision trees in the forest.	16	16	16	5	5	16
Minimum number of samples required to be at a leaf node.	2	4	1	2	4	1
Minimum number of samples required to split an internal node.	2	2	5	10	10	2
Number of decision trees (estimators) in the random forest ensemble	115	45	150	115	10	115

*Optimal configuration of hyperparameters found by grid search. The hyperparameters refer to Table 5 (sensor configuration: accelerometer plus gyroscope plus magnetometer).

Hyperparameters in support vector machine models

To identify the most suitable hyperparameters for the SVM models, a grid search was conducted across a range of hyperparameters, including 'C', 'gamma' and the 'kernel' of the SVM model. C' represents the regularisation parameter, which controls the trade-off between maximising the margin of separation and minimising classification errors. For the grid search, we considered a range of values: 0.1, 1, 10 and 100. Gamma' determines the shape of the decision boundary and plays a crucial role in capturing complex relationships within the data. We chose values of 1, 0.1, 0.01 and 0.001 for 'gamma'. The Radial Basis Function (RBF) was chosen as the kernel for the non-linear classification tasks. To ensure the robustness and reliability of the hyperparameter selection, a cross-validation strategy was employed, with four splits repeated five times.

S4. Model overview and comparison.

Table S8. An overview of all trained machine learning models in this study.

Model	Type	Feature base [a]	Criteria [b]	Movement tasks	Format
Random Forest	Multiclass [c] classification	Euclidean norm (each sensor)	MDS-UPDRS III tasks	AR, OA, FN, HM, PS, FT	Table 4
Support Vector Machine					Table 8
Random Forest	Binary classification	Euclidean norm (each sensor)	Zero versus non-zero MDS-UPDRS III scores	AR, OA, FN, HM, PS, FT	Table 5, including Figure 3 (FN, HM, PS, and FT only)
Support Vector Machine		Euclidean norm (each sensor)			Table 9
Random Forest		Each axis (IMU)			Table 10, including Figure 5 (FN, HM, PS, and FT only)
Support Vector Machine		Each axis (IMU)			Table 12
Random Forest	Multiclass classification	Euclidean norm (each sensor)	Classifying non-zero MDS-UPDRS III scores	FN, HM, PS, FT	Table 6
		Each axis (IMU)			Table 11
Random Forest	Regression	Euclidean norm (each sensor)	MDS-UPDRS III score prediction	FN, HM, PS, FT	Figure 4
		Each axis (IMU)			Figure 6

[a] Specifies the processed signals used for feature calculation.
[b] Indicates the purpose of the classification.
[c] A balanced data set was employed for classifying movement tasks.

Table S9 (Part 1 of 2). Comparison with studies in the literature.

Study reference	Number of participants	Symptom focus [a]	Clinical movement tasks [b]	Study aim	Device/Tool	Senor location	Integrated sensor
Our study	33 PD, 12 controls	Mixed subtype, kinetic-rigid subtype, tremor predominant	AR, OR, FN, HM, PS, and FT assessed by one neurologist [c]	MDS-UPDRS III tasks classification and motor score classification and prediction	miPod V1	Both hands (dorsal part) and intermediate phalanges (index finger)	3-axis accelerometer, 3-axis gyroscope 3-axis magnetometer
Lonini et al. 2018 Ref. **36**	20 PD	Tremor and bradykinesia	Clinical tasks [c] (FT, HM, walking), and daily activities (walking, typing on a keyboard, drawing)	Symptom detection and classification	BioStampRC	Both hands, forearms, and thigh	3-axis accelerometer, 3-axis gyroscope
Shawen et al. 2020 Ref. **13**	13 PD	Tremor and bradykinesia	Walking, typing, pouring water, and FN assessed by one neurologist [c]	Symptom detection and classification	BioStampRC and Apple Watch Series 2	Predominantly affected hand (dorsal part) and wrist	3-axis accelerometer, 3-axis gyroscope
Jeon et al. 2017 Ref. **32**	85 PD	Tremor	AR assessed by two neurologists	Motor score prediction of hand resting tremor	Custom-built sensor device	Wrist and middle finger of left and right hand	3-axis accelerometer, 3-axis gyroscope
Habets et al. 2023 Ref. **19**	37 PD	Bradykinesia	FT assessed by one neurologist	Symptom detection, and finger tapping score prediction	ReTap (custom-built tool)	Distal part of the index finger of left and right hand	3-axis accelerometer

[a] People with PD may experience a variety of symptoms as the disease progresses. The mixed subtype refers to a combination of symptoms, such as tremor, stiffness, and/or slowness of movement (bradykinesia). The akinetic-rigid subtype involves stiffness, bradykinesia, and reduced movement (akinesia). Other symptoms in this list were predominant.

[b] The abbreviations of the movement tasks refer to Table 2 in this article.

Table S9 (Part 2 of 2). Comparison with studies in the literature.

Study reference	Sampling rate	Number of features	Signal processing [a]	Models [b]	Model performance
Our study	50 Hz	13 (and 39)	10-s recordings; segmented into 5-s clips; no overlap; 540 clips; band-pass filtered 0.2 to 20 Hz; sensor magnitude features (and axis features)	SVM, RF	Overall accuracy 0.94 for movement tasks classification (Table 4 and 8). AUROC values 0.68 to 0.92 for motor score classification (Table 5, 6, Table 8 to 12, Figure 3 and 5). Motor score prediction (results in Figure 4 and Figure 6).
Lonini et al. 2018 Ref. **36**	62.5 Hz	10	30-min recordings; segmented into 5-s clips with 50% overlap; 41802 clips; band-pass filtered 0.5 to 3 Hz	RF, CNN	AUROC values (RF and CNN) 0.59 to 0.79 for all sensor combinations, including effects of sensor placement; both symptoms; Figure 2. AUROC values 0.56 to 0.78 (RF and CNN) for symptom detection across activities; both symptoms; Figure 3.
Shawen et al. 2020 Ref. **13**	62.5 Hz (BioStampRC) 50 Hz (Apple Watch)	148	15 to 60 s recordings; segmented into 5-s clips with 50% overlap; 10824 to 16445 clips; high-pass filtered 0.5 Hz	RF	AUROC values 0.63 to 0.79 for all sensor combinations; both symptoms; binary and multiclass; Figure 2 and Table 4.
Jeon et al. 2017 Ref. **32**	125 Hz	13	30-s clips; 170 clips, band-pass filtered 1 to 16 Hz	DT, DA, SVM, kNN	Highest accuracy for decision tree model at 0.85 (RF at 0.83 and SVM at 0.82), Table 4. Number of consensus score {0,1,2,3,4} was {129,38,2,1,0}, Figure 2.
Habets et al. 2023 Ref. **19**	250 Hz	4	10-s clips; 379 clips, bandpass filtered 2 to 48 Hz	DA, SVM, LR, RF	RF model detected tapping blocks in over 94% of cases. Predicted motor scores correlated positively with expert ratings in over 70%. Figure 2 and 3.

[a] Temporal and frequency domain features were extracted from the inertial sensor data in all studies.

[b] Ranked by generalisation ability (i.e., more to less overfitting). Abbreviations: Logistic Regression (LR); Discriminant analysis (DA), Decision trees (DT), K-Nearest Neighbours (kNN), Convolutional neural networks (CNN)
